# β-1,4-Xylan backbone synthesis in higher plants: How complex can it be?

**DOI:** 10.3389/fpls.2022.1076298

**Published:** 2023-01-11

**Authors:** Nadine Anders, Louis Frederick Lundy Wilson, Mathias Sorieul, Nino Nikolovski, Paul Dupree

**Affiliations:** Department of Biochemistry, University of Cambridge, Cambridge, United Kingdom

**Keywords:** Xylan biosynthesis, enzyme complex, primary cell wall, IRX9/IRX9L, IRX10/IRX10L, IRX14/IRX14L

## Abstract

Xylan is a hemicellulose present in the cell walls of all land plants. Glycosyltransferases of the GT43 (IRX9/IRX9L and IRX14/IRX14L) and GT47 (IRX10/IRX10L) families are involved in the biosynthesis of its β-1,4-linked xylose backbone, which can be further modified by acetylation and sugar side chains. However, it remains unclear how the different enzymes work together to synthesize the xylan backbone. A xylan synthesis complex (XSC) has been described in the monocots wheat and asparagus, and co-expression of asparagus *Ao*IRX9, *Ao*IRX10 and *Ao*IRX14A is required to form a catalytically active complex for secondary cell wall xylan biosynthesis. Here, we argue that an equivalent XSC exists for the synthesis of the primary cell wall of the eudicot *Arabidopsis thaliana*, consisting of IRX9L, IRX10L and IRX14. This would suggest the existence of distinct XSCs for primary and secondary cell wall xylan synthesis, reminiscent of the distinct cellulose synthesis complexes (CSCs) of the primary and secondary cell wall. In contrast to the CSC, in which each CESA protein has catalytic activity, the XSC seems to contain proteins with non-catalytic function with each component bearing potentially unique but crucial roles. Moreover, the core XSC formed by a combination of IRX9/IRX9L, IRX10/IRX10L and IRX14/IRX14L might not be stable in its composition during transit from the endoplasmic reticulum to the Golgi apparatus. Instead, potential dynamic changes of the XSC might be a means of regulating xylan biosynthesis to facilitate coordinated deposition of tailored polysaccharides in the plant cell wall.

## Introduction

The plant cell wall is a complex matrix of polysaccharides, proteins and other polymers. The primary cell wall formed around the growing cell mostly consists of cellulose, hemicellulose and pectin, and is generally thin and flexible. By contrast, the secondary cell wall is produced in fully grown specialized cells and forms a thick and more rigid structure with differing polysaccharide composition. Incorporation of lignin rigidifies and reduces the water permeability of the secondary cell wall. Xylan is present in both primary and secondary cell walls (Scheller and Ulvskov, 2010) and is crucial to cell wall strength and biomass recalcitrance. It consists of a linear backbone of β-1,4-linked d-xylosyl residues. The backbone can be substituted with decorations such as acetyl groups, α-d-glucuronic acid, 4-*O*-methyl-α-d-glucuronic acid, α-l-arabinofuranose or β-d-xylose, and the sugar side chains themselves can be further modified. The type, pattern and degree of substitution vary considerably depending on species and tissue, which has been recently reviewed in Smith et al., 2017 and Ye and Zhong, 2022. In addition, differences in primary and secondary xylan structure have been described. In Arabidopsis primary cell wall, glucuronic acid is further substituted with a pentosyl modification, suggested to be α-1,2-l-Ara*p* (Chong et al., 2015; Mortimer et al., 2015). An α-1,2-l-Ara*p* modification on glucuronic acid of xylan was also identified in non-commelinid monocots, though, while present in growing Asparagus tips, it was not detectable in stem xylan (Pena et al., 2016), suggesting that α-1,2-l-Ara*p* modifications on glucuronic acid could be primary cell wall-specific. In contrast to the primary cell wall, the majority of Arabidopsis secondary cell wall xylan carries acetate or glucuronic acid decorations on even-numbered, alternating xylose residues, while other xylan regions have clustered glucuronic acid substitutions (Bromley et al., 2013; Busse-Wicher et al., 2014; Grantham et al., 2017).

Over the years, a plethora of proteins have been implicated in the biosynthesis of xylan. Many are known to modify the xylan backbone or are proposed to produce the essential reducing end oligosaccharide found in eudicots; others are of yet unknown function. An overview of xylan biosynthesis can be found in Smith et al., 2017 and Ye and Zhong, 2022. Here, we would like to focus on three functionally non-redundant groups of proteins that are essential for the β-1,4-xylan backbone synthesis in a wide range of plants (Zhong et al., 2019), comprising two groups assigned to CAZy glycosyltransferase family GT43 (IRX9/IRX9L and IRX14/IRX14L) and one to family GT47 (IRX10/IRX10L) (Drula et al., 2022). Early on, the identification of *irx9* and *irx14* as xylan-deficient Arabidopsis mutants with reduced xylan synthesis activity led to the idea that both enzymes might act together in a protein complex (Brown et al., 2007; Lee et al., 2007; Lee et al., 2012a). More recent genetic evidence supporting the existence of a XSC describes the dominant negative effect of IRX10 point mutants (Brandon et al., 2020). Biochemical analysis of wheat and asparagus proteins further supports the existence of a XSC in monocots (Zeng et al., 2010; Jiang et al., 2016; Zeng et al., 2016). In addition, simultaneous expression of the three asparagus proteins *Ao*IRX9, *Ao*IRX10 and *Ao*IRX14A in tobacco is required to obtain a catalytically active complex in the Golgi.

How three functionally non-redundant putative GTs work together to facilitate the β-1,4-linkage of the xylan backbone, however, remained unanswered. *In vitro* xylosyltransferase or xylan synthase activity has only been successfully demonstrated for isolated GT47 proteins from Arabidopsis, rice, Plantago and Physcomitrium (formerly Physcomitrella), as well as for an IRX10 ortholog from the streptophyte alga *Klebsormidium nitens* (formerly *Klebsormidium flaccidum*) (Jensen et al., 2014; Urbanowicz et al., 2014; Jensen et al., 2018; Wang et al., 2022). The most recent experimental data suggest two *in vitro* activities of rice IRX10: one for elongating the xylan chain and the other for initiating xylan synthesis (Wang et al., 2022), although only elongating activity was detected in Arabidopsis IRX10L (Urbanowicz et al., 2014). Lee et al. showed β-1,4-xylosyltransferase activity of IRX9 and IRX14 when co-expressed in tobacco (Lee et al., 2012a; Lee et al., 2012b)), however, a positive effect of the co-expressed proteins on endogenous IRX10/IRX10L activity cannot be excluded, especially as the activity of IRX9 and IRX14 alone could not be shown (Lee et al., 2012a; Lee et al., 2012b; Urbanowicz et al., 2014).

GT43s adopt a GT-A structural fold; enzymes of this class typically possess an essential catalytic DxD motif, which is critical in coordinating divalent metal ions required for nucleotide-binding (Lairson et al., 2008). Indeed, mutations in the DxD (or DD) motif of *Ao*IRX14A, *Ao*IRX14B and *At*IRX14 result in a loss of function, suggesting that IRX14 could be catalytically active or require UDP-Xyl binding for function (Ren et al., 2014; Zeng et al., 2016). Mutations in the DxD motif of *Ao*IRX9L and *At*IRX9L, however, have no effect on the function of the protein and the motif is not conserved in IRX9. These findings, taken together with the prediction that some IRX10s and IRX10Ls lack a transmembrane domain (Jensen et al., 2014), led to the hypothesis that, rather than a catalytic role, IRX9 and IRX9L might serve a structural role in xylan synthesis (Ren et al., 2014; Zeng et al., 2016). Yet, the functional role of the non-catalytic GTs in the XSC may be more significant in that they could control active complex formation and consequently regulate xylan biosynthesis.

## Evolution of paralogs for primary and secondary cell wall xylan synthesis

Streptophyte algae exhibit both β-1,4-xylan and GT43 xylan synthesis genes. Furthermore, at least for *Klebsormidium nitens*, IRX10 xylan synthesis function has been demonstrated (Taujale and Yin, 2015; Jensen et al., 2018; Hsieh and Harris, 2019). However, in this species, only one ortholog of each group of IRX9/IRX9L, IRX10/IRX10L and IRX14/IRX14L is present. This is also the case in the early vascular model plant *Selaginella moellendorffii*. Gene duplication has taken place in the moss *Physcomitrium patens*, which has only one catalytically active IRX10/IRX10L (Jensen et al., 2014), but two IRX9/IRX9L and three IRX14 paralogs (Hornblad et al., 2013; Haghighat et al., 2016); however, these duplicated genes might not encode functional orthologs of the two paralogous gene groups seen in higher plants. The paralogous genes seen in higher plants emerged at different stages during evolution: the divergence of IRX9 from IRX9L most likely occurred earlier than that of IRX10/IRX10L, or that of IRX14/IRX14L, with separate homologs for IRX9 and IRX9L in monocots and in contrast low sequence diversion of IRX14 and IRX14L in eudicots (Wu et al., 2010; Ren et al., 2014; Wang et al., 2022).

An early step forward in understanding the duplication of xylan biosynthesis enzymes in higher plants came with the realization that each protein is partially redundant with respect to its closest homolog (with the IRX-L mutants displaying less severe phenotypes); the Arabidopsis double mutants of each paralogous pair (*irx9 irx9l, irx10 irx10l* and *irx14 irx14l*) manifest significantly more severe phenotypes than either of the single mutants, but they can functionally replace each other in overexpression and promoter swap experiments (Brown et al., 2009; Wu et al., 2009; Keppler and Showalter, 2010; Lee et al., 2010; Wu et al., 2010; Mortimer et al., 2015). This suggests that differences predominantly arise due to differential expression rather than functional divergence. Supporting this, secondary cell wall xylan in Arabidopsis is mostly dependent on IRX9, IRX10 and IRX14, while IRX9L, IRX10L, and IRX14 are essential for primary cell wall xylan synthesis (Mortimer et al., 2015). Gene expression analyses in asparagus, poplar and rice further support the idea of different sets of IRX homologs being required for xylan biosynthesis in the primary versus the secondary cell wall (Chiniquy et al., 2013; Ratke et al., 2015; Song et al., 2015). The separation into distinct enzyme groups in higher plants is reminiscent of the primary and secondary wall-specific CSCs (Kumar and Turner, 2015).

## The primary cell wall XSC in eudicots

**Figure 1:**
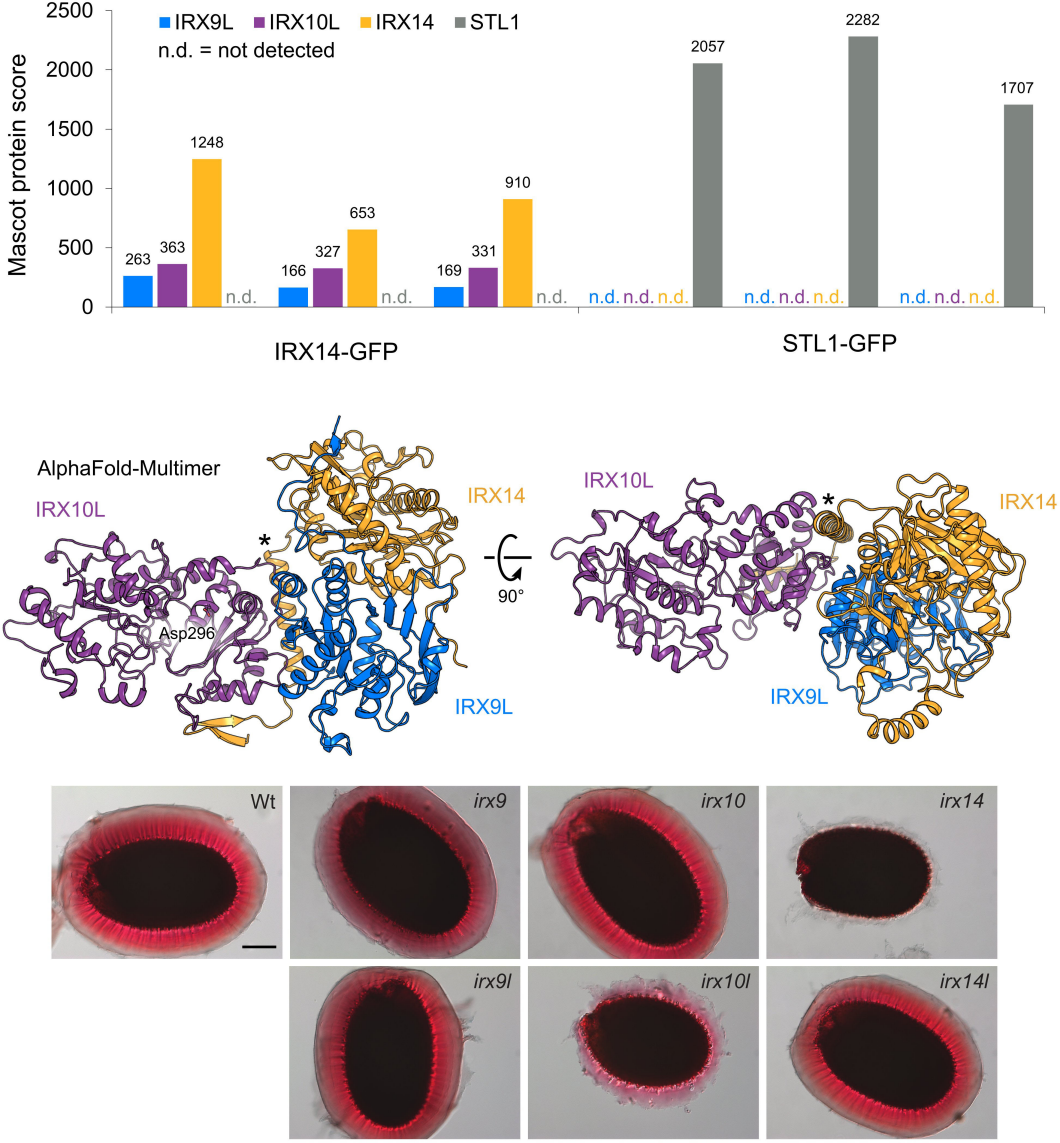
The XSC in primary cell wall xylan synthesis in Arabidopsis. **(A)** Anti-GFP immunoprecipitation of the XSC in Arabidopsis root callus. Mascot protein scores for IRX9L, IRX10L, IRX14 and STL1 (control) are shown for three biological replicates using IRX14-GFP (*left*) or STL1-GFP (control, *right*) as bait, respectively. **(B)** AlphaFold-Multimer model of a heterotrimer of the IRX9L (blue), IRX10L (violet), and IRX14 (yellow) globular domain. A putative nucleotide-binding residue, Asp296 (Wilson et al., 2022), is shown in IRX10L. Note the predicted α-helix of IRX14 at the interaction surfaces of both IRX9L and IRX10L (asterisk). **(C)** Phenotype of the adherent mucilage of XSC mutants. Mucilage is stained with ruthenium red. Wt: Columbia Col-0 wildtype control. Scale bar = 100 µm.

Although research in Arabidopsis has greatly advanced our understanding of xylan synthesis, with genetic data hinting at an interaction of XSC enzymes, protein interaction has not been shown in eudicots. To investigate the existence and composition of a XSC in Arabidopsis, we generated *irx14 irx14l* double-mutant plants expressing IRX14-GFP under its endogenous promoter, creating functional tagged IRX14 (**Supplementary Figure S1**). To investigate XSC formation in primary cell wall-rich tissue, we performed anti-GFP immunoprecipitation in Golgi-enriched microsomal fractions of root callus culture, which we generated from the homozygous transgenic plants. Callus from plants expressing STL1-GFP, a Golgi protein partially co-localizing with IRX9L (Zhang et al., 2016), was used as a negative control. Liquid Chromatography with tandem Mass Spectrometry (LC-MS/MS) analysis was used to detect interacting proteins, showing that IRX14 interacts with IRX9L and IRX10L, but not with STL1 (**Figure 1A**; **Supplementary Table S1**). The immunoprecipitation using STL1-GFP control showed no interaction with IRX9L, IRX10L or IRX14.

These data suggest that IRX9L, IRX10L and IRX14 form a XSC in eudicot primary cell wall-rich tissues, comparable to the XSC synthesizing the secondary cell wall of monocots. Unique peptides were only found for IRX9L and IRX10L, but not for their homologs IRX9 or IRX10 (**Supplementary Table S2**), most likely due to a lack of expression of IRX9 and IRX10 in callus tissue (Mortimer et al., 2015). The detection of IRX9L and IRX10L in the callus XSC is consistent with the genetic evidence that these proteins function together in xylan synthesis of primary cell walls (Mortimer et al., 2015). To support the structural feasibility of a XSC in Arabidopsis, we also note that AlphaFold-Multimer predicts a heterotrimer of the IRX9L, IRX10L and IRX14 globular domains with high intermolecular confidence scores when provided with one copy of each protein (**Figure 1B**; **Supplementary Figure S2A**). The model predicts a pseudo-symmetric interaction of IRX9L and IRX14 and a potential stabilizing role of a conserved proline-rich region/putative α-helix that sits N-terminally of the GT43 domain of IRX14 (**Figure 1B**; **Supplementary Figures S2A, F**). An essentially identical model can be generated from the equivalent secondary cell wall components (**Supplementary Figure S2B**).

## The core of the complex

Genetic and protein interaction studies suggest that xylan is synthesized by a protein complex, however, the protein composition of this complex is not uniformly described. Surprisingly, an IRX9 homolog was not detected in the wheat XSC (Zeng et al., 2010; Jiang et al., 2016), raising the question as to whether all three proteins are required in the active complex.

In *Plantago ovata*, at least four different *IRX10* genes are highly expressed in the seed mucilaginous layer, while IRX9 and IRX14 orthologs are only expressed at very low levels (Jensen et al., 2013; Jensen et al., 2014). Therefore, Jensen et al. (2014) suggested that IRX10 might be solely responsible for mucilage xylan synthesis, whereas in stem tissues of *Plantago*, IRX9 and IRX14 are relevant.

In Arabidopsis, the only known player to synthesize the backbone of seed mucilage xylan is IRX14, whereas, surprisingly, IRX10 appears not to be important (Voiniciuc et al., 2015; Hu et al., 2016). To analyze whether IRX14 is indeed the only XSC protein with a function in xylan biosynthesis in Arabidopsis mucilage, we stained the adherent mucilage of wildtype, *irx9*, *irx9l*, *irx10*, *irx10l*, *irx14* and *irx14l* mutant seeds with ruthenium red. In the absence of xylan, the mucilage does not adhere to the seed (Ralet et al., 2016). The mucilage released from the *irx9*, *irx9l*, *irx10* and *irx14l* mutants had a similar appearance to the mucilage of wildtype seeds, whereas *irx10l* seed mucilage was clearly defective, although the phenotype was less severe than in *irx14* (**Figure 1C**). These data show that IRX10L is also required for xylan biosynthesis in Arabidopsis mucilage. The finding is consistent with the Arabidopsis gene-chip data available on SeedGeneNetwork (Belmonte et al., 2013), showing that IRX10L and IRX14 are highly expressed in the general seed coat at the linear cotyledon stage (the stage of mucilage biosynthesis gene expression; **Supplementary Figure S3A**) (Francoz et al., 2015). An involvement of IRX9/IRX9L in mucilage xylan biosynthesis is unclear due to a lack of an obvious mucilage phenotype in the ruthenium red staining. However, the impact of gene redundancy of IRX9/IRX9L could not easily be assessed, as the double mutants do not produce seeds. Strong expression of IRX9, although possibly delayed to the maturation green stage, might suggest a role of IRX9, whereas IRX9L expression remains low throughout these stages of seed development (**Supplementary Figure S3**).

Taken together, there could be a certain degree of variability even in the three core components of the XSC, depending on species and tissue. Nevertheless, a critical component of xylan synthase activity seems to be IRX10/IRX10L, which is in line with their biochemical activity.

## Homo- and hetero-oligomerization of the XSC components

The apparent size of the asparagus XSCs in native gels is slightly smaller than the marker size of 242 kDa (Zeng et al., 2016). Therefore, the complex could consist of one protein of IRX9/IRX9L, IRX10/IRX10L and IRX14/IRX14L forming a heterotrimeric complex, as shown for the primary and secondary cell wall XSCs in our AlphaFold models (**Figure 1B**; **Supplementary Figures S2A, B**). In tobacco, *Ao*IRX9 and *Ao*IRX14A interact with each other in bimolecular fluorescence complementation experiments (Zeng et al., 2016).

Interestingly, however, the GT43 proteins *Ta*GT43-4, *Ao*IRX9 and *Ao*IRX14A were each shown to interact with themselves (Jiang et al., 2016; Zeng et al., 2016), which is in line with the finding that mammalian GT43 β-1,3-glucuronyltransferases form homodimers (Terayama et al., 1998; Ouzzine et al., 2000). Structural analysis revealed that the three human isoforms homodimerize *via* conserved interaction surfaces (Pedersen et al., 2000; Kakuda et al., 2004; Shiba et al., 2006). As Arabidopsis GT43s are predicted to adopt a similar secondary structure to the human enzymes (Taujale and Yin, 2015), it is possible that homodimerization occurs through similar surfaces. This is supported by AlphaFold modelling, (**Supplementary Figures S2C, D**). This means homodimeric interactions would presumably compete with pseudo-symmetric heterodimerization of IRX9/IRX9L with IRX14/IRX14L by the same interface. However, a trimeric complex of a GT43 homodimer with IRX10L appears less likely, based on the modelling scores. Our AlphaFold modelling of the globular domains alone of two copies of each IRX9L, IRX10L and IRX14 is predicted to form two separate heterotrimers (**Supplementary Figure S2E**). Also, this modelling does not suggest IRX10 dimerization, which is consistent with the results for wheat *Ta*GT47-13 by bimolecular fluorescence complementation (Jiang et al., 2016). On the other hand, bimolecular fluorescence complementation showed self-interaction of asparagus *Ao*IRX10 (Zeng et al., 2016).

In plants, alternative homo- or hetero-oligomerization of GTs has been reported for the *N*-glycan processing Arabidopsis α-mannosidase I and *Nicotiana tabacum* β-1,2-*N*-acetyglucosaminyltransferase I (Schoberer et al., 2013). Another example is the xylosyltransferase XXT2, which can homodimerize, but also interact with XXT5 or XXT1 (Chou et al., 2012). Interestingly, while this homodimerization involves disulfide bridges, heterodimerization seems not to. It is unclear whether plant GT43s only interact *via* the conserved interaction surface of the GT domain, meaning homo- and hetero-oligomerization would be mutually exclusive or whether an additional interaction surface has evolved to allow both simultaneously. Expression in *Pichia pastoris* led to a complex that appeared to contain two *Ta*GT43-4 proteins and one *Ta*GT47-13 (Jiang et al., 2016). This suggests that at least IRX14 can interact with other components of the XSC while forming a homo-oligomer. This was also suggested in the model, proposing a XSC consisting of IRX9 and IRX14 interacting homodimers, which indirectly interact with an IRX10 homodimer (Zeng et al., 2016). In summary, the stoichiometry of the XSC is yet to be understood.

**Figure 2:**
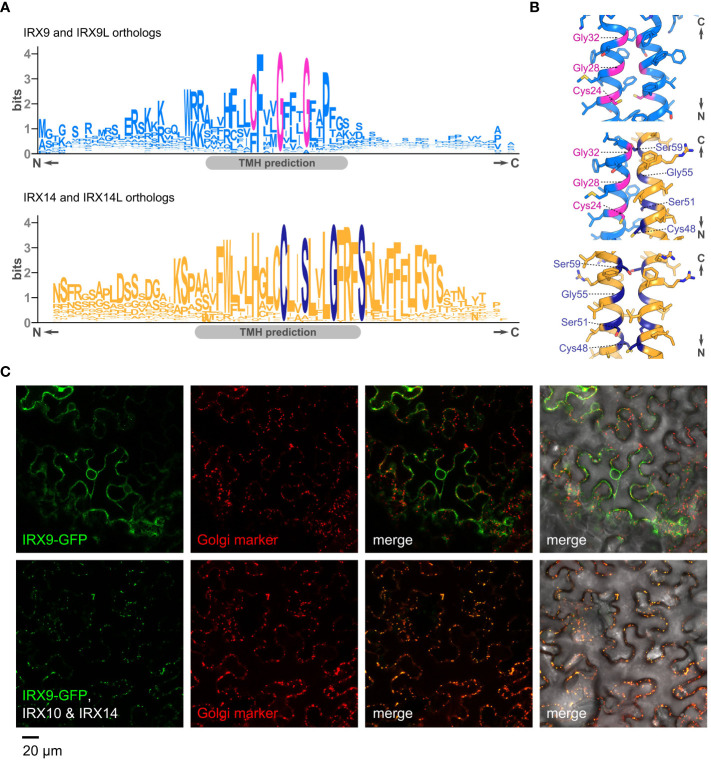
The CTS of GT43s, their potential interactions and impact of interactions on Golgi localization. **(A)**. Sequence logos showing conserved motifs in the predicted transmembrane helices (TMH) of orthologs of IRX9/IRX9L (*top*) and IRX14/IRX14L (*bottom*). **(B)** Close-ups of AlphaFold-Multimer models of the transmembrane dimers for an IRX9 homodimer (*top*), an IRX9–IRX14 heterodimer (*middle*) and an IRX14 homodimer (*bottom*). Residues of the GAS_right_ motif and conserved cysteines are highlighted in magenta or dark blue, respectively. **(C)** Subcellular localization of Arabidopsis IRX9-GFP, transiently expressed in tobacco leaves. Top panel (*left to right*), IRX9-GFP (*green*), ManI-mCherry from soy (Golgi marker, *red*), merge of the two former, and merge including differential interference contrast (DIC) image. Bottom panel shows the same localization analysis in presence of co-expressed, untagged IRX10 and IRX14. Note the change of localization of IRX9-GFP in presence of IRX10 and IRX14.

Golgi GTs are typically type II membrane proteins. Hence, in addition to the luminal GT domain, most exhibit a short cytosolic tail, a single transmembrane domain as well as a stem region (CTS). This CTS region can also mediate protein–protein interactions, including *via* disulfide bridges in the transmembrane helix (TMH) and stem (Tu and Banfield, 2010; Kellokumpu et al., 2016). Interestingly, we found that IRX9/IRX9L orthologs harbor a highly conserved CFxxGxxxG motif in their predicted TMH (**Figure 2A**), resembling a canonical GxxxG motif, which could act as a GAS_right_ helix oligomerization motif like that in glycophorin A (Mueller et al., 2014). The previously identified WxxxHxxCCxxSxxLGxRFS motif of IRX14/IRX14L orthologs (Jiang et al., 2016) could be considered a GAS_right_ variant since it contains a SxxxGxxxS motif (**Figure 2A**). Indeed, AlphaFold modelling supports the notion that the IRX9 and IRX14 TMHs could form a disulfide-linked homo- or heterodimer with the GAS_right_ motifs situated at the helix interface (**Figure 2B**), providing a potential mechanism of interaction. This motif might provide an additional dimerization surface, allowing larger asparagus XSC complex formation such as that additionally detected in the native gel analysis running just below the 480kDa marker (Zeng et al., 2016).

## Subcellular localization of the XSC and dynamic changes of its composition

As well as mediating protein interactions, the CTS region of Golgi GTs can determine correct localization to distinct Golgi cisternae (Schoberer and Strasser, 2011; Schoberer et al., 2013). The cytoplasmic tail of IRX14 and IRX14L also contains a conserved cytosolic di-arginine motif (Jiang et al., 2016), a motif that has been described in governing ER retrieval or Golgi localization (Welch and Munro, 2019). Proteins in assembled complexes with an ER-retaining motif can pass to the Golgi, possibly through masking of the motif (Michelsen et al., 2005; Banfield, 2011). Ren et al., 2014 noted that the milder phenotype of *irx9-2* (versus *irx9-1*) might be due to the presence of the truncated protein, assigning a potential function to the N-terminus of IRX9.

Notably, the interaction of *Ta*GT47-13 and *Ta*GT43-4 already occurs in the ER and their co-expression is required for ER to Golgi translocation of the complex in tobacco (Jiang et al., 2016). Using a similar approach, co-expression of *Ao*IRX9, *Ao*IRX10 and *Ao*IRX14A was shown to be required for catalytic activity and XSC transition from the ER to the Golgi (Zeng et al., 2016). This mechanism seems not unique to monocots; we have also observed that co-expression of IRX10 and IRX14 is required for the Golgi-localization of IRX9-GFP in tobacco (**Figure 2C**). Co-expression with IRX10 or IRX14 alone were not tested.

Sub-Golgi localization of GTs in Arabidopsis using free-flow electrophoresis followed by mass-spectrometry analysis revealed that IRX14 and the glucuronyltransferase GUX3 mostly locate to the *trans*-Golgi, while IRX10L was predominantly found in *medial*-Golgi (Parsons et al., 2019). Quantitative immuno-transmission electron microscopy also shows that IRX9 localizes predominantly to the *medial-*Golgi (Meents et al., 2019). Hence, the three core components of the XSC seem not consistently co-localized, suggesting they do not always exist in the same complex. Similar to the sequential distribution of GTs involved in *N*-glycosylation of proteins, recent models suggest that GTs involved in polysaccharide biosynthesis of the plant cell wall are distributed in different Golgi-cisternae *cis, medial* to *trans*, depending on their functional activity (Parsons et al., 2019; Hoffmann et al., 2021). Supporting this idea is the reported absence of IRX9 and presence of two putative UDP-sugar mutases, *Ta*GT75-3 and *Ta*GT75-4, and two non-GTs, *Ta*GLP and *Ta*VER2, in the wheat XSC (Zeng et al., 2010; Jiang et al., 2016), although these interactions have not been described in other systems. The XSC isolated from Golgi-enriched membranes of wheat also exhibited arabinosyl- and glucuronyltransferase activity, indicating that the XSC might harbor xylan modifying enzymes, although the respective enzymes were not detected (Zeng et al., 2010; Jiang et al., 2016). Similarly, in our immunoprecipitation study we did not detect co-precipitation of the glucuronyltransferase GUX3. Nevertheless, it is tempting to think that a specialized primary cell wall XSC might differ in its interactions compared with the secondary cell wall XSC, leading to the differences reported in GlcA modification patterns of xylan (Mortimer et al., 2010; Bromley et al., 2013). The interaction of the core XSC proteins with xylan backbone-modifying enzymes would be expected to occur in the *trans*-Golgi. Other potential interactors (for example the UDP-Xyl transporter UXT1, which is suggested to channel the UDP-Xyl substrate for xylan biosynthesis (Ebert et al., 2015)) might interact with the XSC early in the Golgi. Hence, the XSC might not have a uniform composition, but rather might change while transiting from the ER to the Golgi and through the Golgi cisternae, reflecting its functional activity.

## Discussion

All three components of the XSC, IRX9/9L, IRX14/IRX14L and IRX10/IRX10L, are necessary for xylan synthesis in most systems. Despite this, the emerging picture is that IRX10/IRX10L is the catalytically active enzyme essential for the biochemical function of the XSC. In contrast, the role of IRX9/9L and IRX14/IRX14L in the XSC remains poorly understood and future research will have to establish why IRX9/IRX9L and IRX14/IRX14L are essential for xylan synthesis, albeit not directly involved in the catalytic reaction. Diverse non-catalytic functions of the GT-like proteins have been suggested over the years. These range from membrane anchoring of or UDP-Xyl channeling to IRX10/IRX10L, to serving as a scaffold for XSC assembly or assembly-dependent trafficking (Ren et al., 2014; Jiang et al., 2016; Zeng et al., 2016).

In mammalian Golgi *N*-glycosyltransferases, homomers are disassembled and heteromers formed depending on pH-changes during transition through the Golgi (Hassinen et al., 2010; Hassinen and Kellokumpu, 2014). It is unknown whether such a mechanism plays a role in plants; however, it is important to keep in mind that interactions between proteins might not be static. Thus, one step to understanding the role of the individual XSC components might be through analysis of their sub-Golgi localization, complex formation and interaction with xylan-modifying enzymes. Finally, changes in the protein composition of the XSC through homo- and heterodimerization of its components could provide a mechanistic tool to regulate the localization of the XSC and its activity in response to environmental cues, allowing dynamic adjustment of the plant cell wall.

## Data availability statement

The original contributions presented in the study are included in the article/**Supplementary Material**. Further inquiries can be directed to the corresponding author.

## Author contributions

MS created transgenic lines, NN and MS performed immunoprecipitation analysis; NN, NA, and MS performed the mucilage staining. LW conducted transient expression in tobacco, sequence analysis, and AlphaFold modelling. NA conceptualized the manuscript, collated the data and wrote the manuscript with support of LW and PD. All authors contributed to the article and approved the submitted version.
