# Supplementary material for: β-1,4-Xylan backbone synthesis in higher plants: How complex can it be?
Source: Front Plant Sci. 2023 Jan 11;13:1076298. doi: 10.3389/fpls.2022.1076298 (PMC9874913; doi:10.3389/fpls.2022.1076298)
Supplement: Supplementary file 1 [file DataSheet_1.docx]

**Supplementary Materials and Methods**

**Plant material and Plant Growth Conditions**

The T-DNA insertion lines used were *irx9* (SALK_ 057033), *irx9l* (SALK_037323), *irx10* (SALK_046368), *irx10l* (GK-179G11), *irx14* (SALK_038212) and *irx14l* (SALK_066961) (Mortimer et al., 2015). Arabidopsis seeds were either sown out on soil or surface sterilized and sown onto solid medium (0.5 × Murashige and Skoog salts, 1% sucrose (w/v), 0.8% Plant agar pH 5.8). Seeds were stratified in the dark for 48 h at 4**°**C and transferred to a growth room (20°C, 100 μmol m^−2^ s^−1^, 16 h light/8 h dark, 60% humidity). After 1–2 weeks, seedlings on plates were transferred to soil.

*Arabidopsis thaliana* root callus cultures were established from the roots of transgenic seedlings as described (Sherrier et al., 1999). STL1-GFP root callus culture (Zhang et al., 2016) was used as control. The callus was harvested 4 days after refreshing the growth medium.

**Cloning of *_pro_IRX14:IRX14-GFP* and generation of transgenic lines**

Vector: The PCR-amplified expression cassette consisting of the oleosin 1 (OLE1) promoter *OLE1-GFP* and 35S terminator from pFAST-G01 (Shimada et al., 2010) was inserted into the *Kpn*I and *Apa*I restriction site of pGreenII0000 to use fluorescence of dry seeds as selection marker. The NOS terminator was inserted into the *Not*I and *Sac*I sites.

*IRX14-mGFP*: The genomic region of *IRX14*, with an upstream promoter region of 1.52 kb, was PCR amplified from genomic DNA of *Arabidopsis thaliana* Col-0 ecotype. The primers added the restriction site *Apa*I to the 5' and *Cla*I/*Pst*I to the 3' end of the PCR product (concomitantly removing the stop codon at the 3’end). The product was inserted into the vector using *Apa*I and *Pst*I*.* The primers for the mGFP PCR product added restrictions sites *Cla*I/*Pst*I to the 5' and *Not*I restriction site to the 3' end. Insertion into the vector used *Pst*I and *Not*I restriction sites.

Arabidopsis homozygous *irx14* and heterozygous *irx14l* mutant plants, were transformed with *_pro_IRX14:IRX14-mGFP*. The plants were transformed using *Agrobacterium tumefaciens* (GV3101) via the floral dipping method (Clough and Bent, 1998). At least three T3 homozygous mono-insertional independent transgenic lines were selected using OLE1-GFP as seed selection marker. The analysis was conducted on complemented double mutant *irx14 irx14l*.

**Transient expression in *Nicotiana benthamiana* and confocal analysis**

Coding sequences for Arabidopsis IRX9, IRX10, IRX14, and soy mannosidase I (ManI_1–59_) were synthesized *de novo* complete with *Bsa*I restriction sites compatible with Plant MoClo cloning. IRX9 was cloned with a C-terminal GFP tag, whereas IRX10 and IRX14 were each cloned with C-terminal 3×Myc tag. Constructs were assembled using the OpenPlant MoClo toolkit (Engler et al., 2014;Patron et al., 2015). All coding sequences were placed under the 35S promoter and terminated with the cow pea mosaic virus (CPMV) 5’ UTR / terminator from pEAQ-HT.

*Nicotiana benthamiana* leaves were infiltrated with one plasmid harbouring ManI_1–59_-mCherry and IRX9-GFP, or a plasmid harbouring both proteins as well as Myc-tagged IRX10 and IRX14. The transient expression was carried out as described by Sparkes et al (Sparkes et al., 2006) using *Agrobacterium tumefaciens* strain AGL1. Confocal images were acquired three days post infiltration using a Leica SP8 laser scanning confocal microscope with a 63× water objective. GFP and mCherry fluorophores were excited with 488 and 561 nm-wavelength lasers, respectively.

**Microsome Protein Preparation and Immunoprecipitation**

Root callus was harvested and detergent soluble membranes prepared at 4 °C. Approximately 50 g of callus were homogenized in 50 ml of homogenization buffer (250 mM sucrose, 25 mM HEPES, pH 7.5, 1 mM EDTA, 1 mM dithiothreitol, and cOmplete^TM^ Protease Inhibitor Cocktail Tablets). The homogenate was filtered with a nylon mesh (45 µm) and centrifuged twice for 30 min at 3200 × *g* to remove cell debris. The supernatant was centrifuged for 1 h at 4°C in an SW32 rotor at 175,000 × *g* onto a 6-ml cushion containing 10% (v/v) iodixanol in homogenization buffer. The interphase was collected, pelleted for 20 min at 175,000 × *g* and resuspended in 2 ml of immunoprecipitation buffer (25 mM HEPES-KOH, pH 7.5; 150 mM NaCl; 1 mM PMSF; cOmplete^TM^ protease inhibitors; 1% triton X100 (v/v)). The samples were slowly inverted for 30 min and then centrifuged for 20 min at 20,000 × *g*. The supernatant representing the detergent soluble membranes was used for immunoprecipitation with GFP-Trap® (gtma-20, ChromoTek).

Equilibrated agarose beads coated with antibody were added (20 µl of beads slurry per 1 mg of protein) to the supernatant and incubated vertically rotating for 16 h. The beads were then washed three times with immunoprecipitation buffer and then eluted with glycine pH 2.

90% of the eluate was loaded on 4–15% gradient SDS-PAGE gels and run for 15 min at 100 V. The gel lanes were sliced into 1-mm cubes and dehydrated with 50% acetonitrile in 50mM ammonium formate. The protein samples were reduced with 5 mM DTT for 1 h at 65 °C and alkylated with 15 mM iodoacetamide for 1 h at room temperature in the dark. The samples were digested with 5 μg MS-grade modified trypsin (Promega) for 16 h at 37°C. The peptide products were collected from the samples and 0.5% (v/v) trifluoroacetic acid and 3% (v/v) acetonitrile was added to the liquid before injection into the Liquid Chromatography with tandem Mass Spectrometry (LC-MS/MS) system.

**LC-MS/MS and data analysis**

All LC-MS/MS experiments were performed using a Dionex Ultimate 3000 RSLC nanoUPLC (Thermo Fisher Scientific Inc, Waltham, MA, USA) system and a QExactive Orbitrap mass spectrometer (Thermo Fisher Scientific Inc, Waltham, MA, USA). Separation of peptides was performed by reverse-phase chromatography at a flow rate of 300 nl min^−1^ and a Thermo Scientific reverse-phase nano Easy-spray column (Thermo Scientific PepMap C18, 2-mm particle size, 100A pore size, 75 mm i.d. × 50 cm length). Peptides were loaded onto a pre-column (Thermo Scientific PepMap 100 C18, 5-mm particle size, 100A pore size, 300 mm i.d. × 5mm length) from the Ultimate 3000 autosampler with 0.1% formic acid for 3 min at a flow rate of 10 ml min^−1^. After this period, elution of peptides from the pre-column onto the analytical column was allowed using Solvent A (0.1% formic acid) and solvent B (80% acetonitrile, 20% water and 0.1% formic acid solution). The linear gradient employed was 2–30% B in 30 minutes. The LC eluent was sprayed into the mass spectrometer by means of an Easy-spray source (Thermo Fisher Scientific Inc.). All m/z values of eluting ions were measured in an Orbitrap mass analyzer, set at a resolution of 70,000 (FWHM) at m/z 200. Data dependent scans (Top 20) were employed to automatically isolate precursors and generate fragment ions by higher-energy collisional dissociation in the quadrupole mass analyzer. Measurement of the resulting fragment ions was performed in the Orbitrap analyzer, set at a resolution of 17,500. Peptide ions with charge states of +2 and above were selected for fragmentation.

The MS data were processed by converting the raw data files into the mgf format. The mgf files were searched against the nrTAIR10 database (in-house built non-redundant TAIR10 database) using Mascot Daemon version V2.3.2. by Matrix Science. The mass accuracy of the precursor ions was set to 1 Da and the fragment mass accuracy was set to 0.5 Da. The considered amino acid modifications were set for variable modification of methionine oxidation and fixed modification of carbamidomethyl cysteine. Two missed cleavages for trypsin were permitted. The Mascot protein scores, with a Mascot peptide significance threshold *p* < 0.05 applied, were extracted from the output reports.

**Mucilage staining**

Around 50 *Arabidopsis thaliana* seeds were incubated for 2 h in water and then stained with a 0.01 % (w/v) Ruthenium red solution for 30 min. They were observed with a 20x objective on a Zeiss Axioimager M2 microscope using high quality differential interference contrast (DIC) imaging.

**DNA sequencer-Assisted Saccharide analysis in High throughput (DASH)**

Basal stem from *_pro_IRX14:IRX14-GFP* *irx14 irx14l* plants were harvested and alcohol insoluble residue (AIR) was prepared (Tryfona et al., 2019). AIR (100 μg) was treated with 4 M NaOH for 1 h at room temperature before adjusting the pH to pH 5–6 with 1 M HCl and hydrolysed with the glucuronoxylanase *Bo*GH30 (Rogowski et al., 2015) in 500 μl 0.1 M ammonium acetate, pH 6.0. The mixture was boiled and the oligosaccharides were labelled with aminopyrene trisulfonic acid (APTS) and analyzed in a DNA sequencer (Li et al., 2013;Tryfona et al., 2019). DASH data were processed in DASHboard software. Xylose standards: xylose (X), X_2_, X_3_, X_4_, X_5_ and X_6_ were APTS labelled and analysed in parallel. Nomenclature of xylan oligosaccharides is according to Faure et al., 2009 (Faure et al., 2009).

**Sequence alignment and logo generation of GT43s**

For the alignment of the IRX14/IRX14L sequences, GT43 sequences were downloaded from the PlantCAZyme database (Ekstrom et al., 2014). A GT43 hidden Markov model constructed using HMMER (Eddy, 2008) was used to extract further GT43 sequences from proteome models from *Klebsormidium nitens* (Hori et al., 2014), *Mesotaenium endlicheranium* (Cheng et al., 2019), *Anthoceros angustus* (Zhang et al., 2020) and *Salvinia cucullata* (Li et al., 2018). An alignment using MAFFT (Katoh et al., 2002) and a phylogeny using FastTree (Price et al., 2010) was then inferred to sort IRX14/IRX14 orthologs from IRX9/IRX9L orthologs. IRX14/IRX14L orthologs from the selected species were then extracted and re-aligned using MUSCLE (Edgar, 2004a;b). In total 76 IRX14/IRX14L orthologs and 163 IRX9/IRX9L orthologs were aligned.

To create the transmembrane helix (TMH) alignment, TMHMM (Krogh et al., 2001) was used to find the locations of all potential TMHs in the full set of GT43 sequences. A bespoke Python script was then used to extract the putative TMHs along with the twenty adjacent residues on each side. The extracted sequences were then aligned with MUSCLE. The gap opening penalty was set to −30 for IRX9/IRX9L and −15 for IRX14/IRX14L sequences to prevent the formation of gaps in the transmembrane region. The alignment was transformed into a sequence logo using WebLogo 2.8.2 (Crooks et al., 2004).

**AlphaFold-Multimer**

For the modelling of the XSC, sequences corresponding to the globular domains of IRX9 (residues 113–352), IRX9L (residues 139–394), IRX10 (residues 27–412) IRX10L (residues 29–415) and IRX14 (residues 113–443) were used; for the modelling of the transmembrane dimers, sequences corresponding to the N-terminus of IRX9 (residues 1–49) and IRX14 (1–85) were used and input into the online AlphaFold (v2.2) (Jumper et al., 2021;Evans et al., 2022) Colab notebook (<https://colab.research.google.com/github/deepmind/alphafold/blob/main/notebooks/AlphaFold.ipynb>). The predicted aligned error plot was re-plotted using a bespoke Python script. Structural models were rendered in PyMOL.

**Supplemental References**

Cheng, S., Xian, W., Fu, Y., Marin, B., Keller, J., Wu, T., Sun, W., Li, X., Xu, Y., Zhang, Y., Wittek, S., Reder, T., Gunther, G., Gontcharov, A., Wang, S., Li, L., Liu, X., Wang, J., Yang, H., Xu, X., Delaux, P.M., Melkonian, B., Wong, G.K., and Melkonian, M. (2019). Genomes of Subaerial Zygnematophyceae Provide Insights into Land Plant Evolution. *Cell* 179**,** 1057-1067 e1014.

Clough, S.J., and Bent, A.F. (1998). Floral dip: a simplified method for Agrobacterium-mediated transformation of Arabidopsis thaliana. *Plant J* 16**,** 735-743.

Crooks, G.E., Hon, G., Chandonia, J.M., and Brenner, S.E. (2004). WebLogo: a sequence logo generator. *Genome Res* 14**,** 1188-1190.

Eddy, S.R. (2008). A probabilistic model of local sequence alignment that simplifies statistical significance estimation. *PLoS Comput Biol* 4**,** e1000069.

Edgar, R.C. (2004a). MUSCLE: a multiple sequence alignment method with reduced time and space complexity. *BMC Bioinformatics* 5**,** 113.

Edgar, R.C. (2004b). MUSCLE: multiple sequence alignment with high accuracy and high throughput. *Nucleic Acids Res* 32**,** 1792-1797.

Ekstrom, A., Taujale, R., Mcginn, N., and Yin, Y. (2014). PlantCAZyme: a database for plant carbohydrate-active enzymes. *Database (Oxford)* 2014.

Engler, C., Youles, M., Gruetzner, R., Ehnert, T.M., Werner, S., Jones, J.D., Patron, N.J., and Marillonnet, S. (2014). A golden gate modular cloning toolbox for plants. *ACS Synth Biol* 3**,** 839-843.

Evans, R., O’neill, M., Pritzel, A., Antropova, N., Senior, A., Green, T., Žídek, A., Bates, R., Blackwell, S., Yim, J., Ronneberger, O., Bodenstein, S., Zielinski, M., Bridgland, A., Potapenko, A., Cowie, A., Tunyasuvunakool, K., Jain, R., Clancy, E., Kohli, P., Jumper, J., and Hassabis, D. (2022). Protein complex prediction with AlphaFold-Multimer. *bioRxiv*.

Faure, R., Courtin, C.M., Delcour, J.A., Dumon, C., Faulds, C.B., Fincher, G.B., Fort, S., Fry, S.C., Halila, S., Kabel, M.A., Pouvreau, L., Quemener, B., Rivet, A., Saulnier, L., Schols, H.A., Driguez, H., and O'donohue, M.J. (2009). A Brief and Informationally Rich Naming System for Oligosaccharide Motifs of Heteroxylans Found in Plant Cell Walls. *Australian Journal of Chemistry* 62**,** 533-537.

Hori, K., Maruyama, F., Fujisawa, T., Togashi, T., Yamamoto, N., Seo, M., Sato, S., Yamada, T., Mori, H., Tajima, N., Moriyama, T., Ikeuchi, M., Watanabe, M., Wada, H., Kobayashi, K., Saito, M., Masuda, T., Sasaki-Sekimoto, Y., Mashiguchi, K., Awai, K., Shimojima, M., Masuda, S., Iwai, M., Nobusawa, T., Narise, T., Kondo, S., Saito, H., Sato, R., Murakawa, M., Ihara, Y., Oshima-Yamada, Y., Ohtaka, K., Satoh, M., Sonobe, K., Ishii, M., Ohtani, R., Kanamori-Sato, M., Honoki, R., Miyazaki, D., Mochizuki, H., Umetsu, J., Higashi, K., Shibata, D., Kamiya, Y., Sato, N., Nakamura, Y., Tabata, S., Ida, S., Kurokawa, K., and Ohta, H. (2014). Klebsormidium flaccidum genome reveals primary factors for plant terrestrial adaptation. *Nat Commun* 5**,** 3978.

Jumper, J., Evans, R., Pritzel, A., Green, T., Figurnov, M., Ronneberger, O., Tunyasuvunakool, K., Bates, R., Zidek, A., Potapenko, A., Bridgland, A., Meyer, C., Kohl, S.a.A., Ballard, A.J., Cowie, A., Romera-Paredes, B., Nikolov, S., Jain, R., Adler, J., Back, T., Petersen, S., Reiman, D., Clancy, E., Zielinski, M., Steinegger, M., Pacholska, M., Berghammer, T., Bodenstein, S., Silver, D., Vinyals, O., Senior, A.W., Kavukcuoglu, K., Kohli, P., and Hassabis, D. (2021). Highly accurate protein structure prediction with AlphaFold. *Nature* 596**,** 583-589.

Katoh, K., Misawa, K., Kuma, K., and Miyata, T. (2002). MAFFT: a novel method for rapid multiple sequence alignment based on fast Fourier transform. *Nucleic Acids Res* 30**,** 3059-3066.

Krogh, A., Larsson, B., Von Heijne, G., and Sonnhammer, E.L. (2001). Predicting transmembrane protein topology with a hidden Markov model: application to complete genomes. *J Mol Biol* 305**,** 567-580.

Li, F.W., Brouwer, P., Carretero-Paulet, L., Cheng, S., De Vries, J., Delaux, P.M., Eily, A., Koppers, N., Kuo, L.Y., Li, Z., Simenc, M., Small, I., Wafula, E., Angarita, S., Barker, M.S., Brautigam, A., Depamphilis, C., Gould, S., Hosmani, P.S., Huang, Y.M., Huettel, B., Kato, Y., Liu, X., Maere, S., Mcdowell, R., Mueller, L.A., Nierop, K.G.J., Rensing, S.A., Robison, T., Rothfels, C.J., Sigel, E.M., Song, Y., Timilsena, P.R., Van De Peer, Y., Wang, H., Wilhelmsson, P.K.I., Wolf, P.G., Xu, X., Der, J.P., Schluepmann, H., Wong, G.K., and Pryer, K.M. (2018). Fern genomes elucidate land plant evolution and cyanobacterial symbioses. *Nat Plants* 4**,** 460-472.

Li, X., Jackson, P., Rubtsov, D.V., Faria-Blanc, N., Mortimer, J.C., Turner, S.R., Krogh, K.B., Johansen, K.S., and Dupree, P. (2013). Development and application of a high throughput carbohydrate profiling technique for analyzing plant cell wall polysaccharides and carbohydrate active enzymes. *Biotechnol Biofuels* 6**,** 94.

Mortimer, J.C., Faria-Blanc, N., Yu, X., Tryfona, T., Sorieul, M., Ng, Y.Z., Zhang, Z., Stott, K., Anders, N., and Dupree, P. (2015). An unusual xylan in Arabidopsis primary cell walls is synthesised by GUX3, IRX9L, IRX10L and IRX14. *Plant J* 83**,** 413-426.

Patron, N.J., Orzaez, D., Marillonnet, S., Warzecha, H., Matthewman, C., Youles, M., Raitskin, O., Leveau, A., Farre, G., Rogers, C., Smith, A., Hibberd, J., Webb, A.A., Locke, J., Schornack, S., Ajioka, J., Baulcombe, D.C., Zipfel, C., Kamoun, S., Jones, J.D., Kuhn, H., Robatzek, S., Van Esse, H.P., Sanders, D., Oldroyd, G., Martin, C., Field, R., O'connor, S., Fox, S., Wulff, B., Miller, B., Breakspear, A., Radhakrishnan, G., Delaux, P.M., Loque, D., Granell, A., Tissier, A., Shih, P., Brutnell, T.P., Quick, W.P., Rischer, H., Fraser, P.D., Aharoni, A., Raines, C., South, P.F., Ane, J.M., Hamberger, B.R., Langdale, J., Stougaard, J., Bouwmeester, H., Udvardi, M., Murray, J.A., Ntoukakis, V., Schafer, P., Denby, K., Edwards, K.J., Osbourn, A., and Haseloff, J. (2015). Standards for plant synthetic biology: a common syntax for exchange of DNA parts. *New Phytol* 208**,** 13-19.

Price, M.N., Dehal, P.S., and Arkin, A.P. (2010). FastTree 2--approximately maximum-likelihood trees for large alignments. *PLoS One* 5**,** e9490.

Rogowski, A., Briggs, J.A., Mortimer, J.C., Tryfona, T., Terrapon, N., Lowe, E.C., Basle, A., Morland, C., Day, A.M., Zheng, H., Rogers, T.E., Thompson, P., Hawkins, A.R., Yadav, M.P., Henrissat, B., Martens, E.C., Dupree, P., Gilbert, H.J., and Bolam, D.N. (2015). Glycan complexity dictates microbial resource allocation in the large intestine. *Nat Commun* 6**,** 7481.

Sherrier, D.J., Prime, T.A., and Dupree, P. (1999). Glycosylphosphatidylinositol-anchored cell-surface proteins from Arabidopsis. *Electrophoresis* 20**,** 2027-2035.

Shimada, T.L., Shimada, T., and Hara-Nishimura, I. (2010). A rapid and non-destructive screenable marker, FAST, for identifying transformed seeds of Arabidopsis thaliana. *Plant J* 61**,** 519-528.

Sparkes, I.A., Runions, J., Kearns, A., and Hawes, C. (2006). Rapid, transient expression of fluorescent fusion proteins in tobacco plants and generation of stably transformed plants. *Nat Protoc* 1**,** 2019-2025.

Tryfona, T., Sorieul, M., Feijao, C., Stott, K., Rubtsov, D.V., Anders, N., and Dupree, P. (2019). Development of an oligosaccharide library to characterise the structural variation in glucuronoarabinoxylan in the cell walls of vegetative tissues in grasses. *Biotechnol Biofuels* 12**,** 109.

Zhang, J., Fu, X.X., Li, R.Q., Zhao, X., Liu, Y., Li, M.H., Zwaenepoel, A., Ma, H., Goffinet, B., Guan, Y.L., Xue, J.Y., Liao, Y.Y., Wang, Q.F., Wang, Q.H., Wang, J.Y., Zhang, G.Q., Wang, Z.W., Jia, Y., Wang, M.Z., Dong, S.S., Yang, J.F., Jiao, Y.N., Guo, Y.L., Kong, H.Z., Lu, A.M., Yang, H.M., Zhang, S.Z., Van De Peer, Y., Liu, Z.J., and Chen, Z.D. (2020). The hornwort genome and early land plant evolution. *Nat Plants* 6**,** 107-118.

Zhang, Y., Nikolovski, N., Sorieul, M., Vellosillo, T., Mcfarlane, H.E., Dupree, R., Kesten, C., Schneider, R., Driemeier, C., Lathe, R., Lampugnani, E., Yu, X., Ivakov, A., Doblin, M.S., Mortimer, J.C., Brown, S.P., Persson, S., and Dupree, P. (2016). Golgi-localized STELLO proteins regulate the assembly and trafficking of cellulose synthase complexes in Arabidopsis. *Nat Commun* 7**,** 11656.

**Supplementary Figure Legends**

**Figure S1**

**A** Complementation of *irx14 irx14l* with *_pro_IRX14:IRX14-GFP*. Plant phenotype (*top*) of the Col-0 wildtype control (*left*), *irx14/irx14l* (*middle*) and *_pro_IRX14:IRX14-GFP* in *irx14/irx14l* (*right*). **B** DASH traces of the GH30 enzyme digest of xylan AIR. Xylose (X) standards (X_3_-X_6_) are shown (*top*), *_pro_IRX14:IRX14-GFP* in *irx14/irx14l* (*middle*) and wildtype (*bottom*). Double peaks seen in WT and complemented plants reflect the unmethylated xylooligosaccharide modified with glucuronic acid (U): UX (smaller peak) and methylated U^m^X (larger peak) of the respective oligosaccharide.

**Figure S2**

AlphaFold-Multimer models of the globular domains of XSC proteins with predicted aligned error plots. Greener ‘cross-peaks’ in the predicted aligned error plots, indicate higher confidence in the relative position of the aligned residue with respect to the scored residue. **A** Primary cell wall XSC heterotrimer consisting of one copy of IRX9L, IRX10L and IRX14. **B** Secondary cell wall XSC heterotrimer consisting of one copy of IRX9, IRX10 and IRX14. Note, the similarity between the two models. **C** Model of the interaction of the IRX9L homodimer with one copy of IRX10L. **D** Model of the interaction of the IRX14 homodimer with one copy of IRX10L. Note, the lower confidence scores in the interaction of the homodimer with IRX10L. **E** Model of two copies of IRX9L, IRX10L and IRX14, resulting in two heterotrimeric complexes; Amber relaxation was disabled for model E to prevent crashing. **F** Section of an alignment of IRX14/IRX14L orthologs from a wide range of streptophytes showing the proline-rich region and predicted α-helix preceding the GT43 domain. Particularly well conserved residues are highlighted in bold, and the predicted secondary structure is indicated below the alignment.

**Figure S3**

Gene expression of XSC proteins in laser capture micro-dissected general seed coat at linear-cotyledon stage (**A**) and mature green stage (**B**). The two columns shown for each gene represent the signal intensity of two biological replicates of Arabidopsis Ws-0 analysed with the GeneChip ATH1 Arabidopsis Genome Array. Asterisks mark values not present in both replicates (marked as marginal or absent in the original study). Note the different Y-axis scale of the graphs.
