# Supplementary figures and images for: β-1,4-Xylan backbone synthesis in higher plants: How complex can it be?
Source: Front Plant Sci. 2023 Jan 11;13:1076298. doi: 10.3389/fpls.2022.1076298 (PMC9874913; doi:10.3389/fpls.2022.1076298)

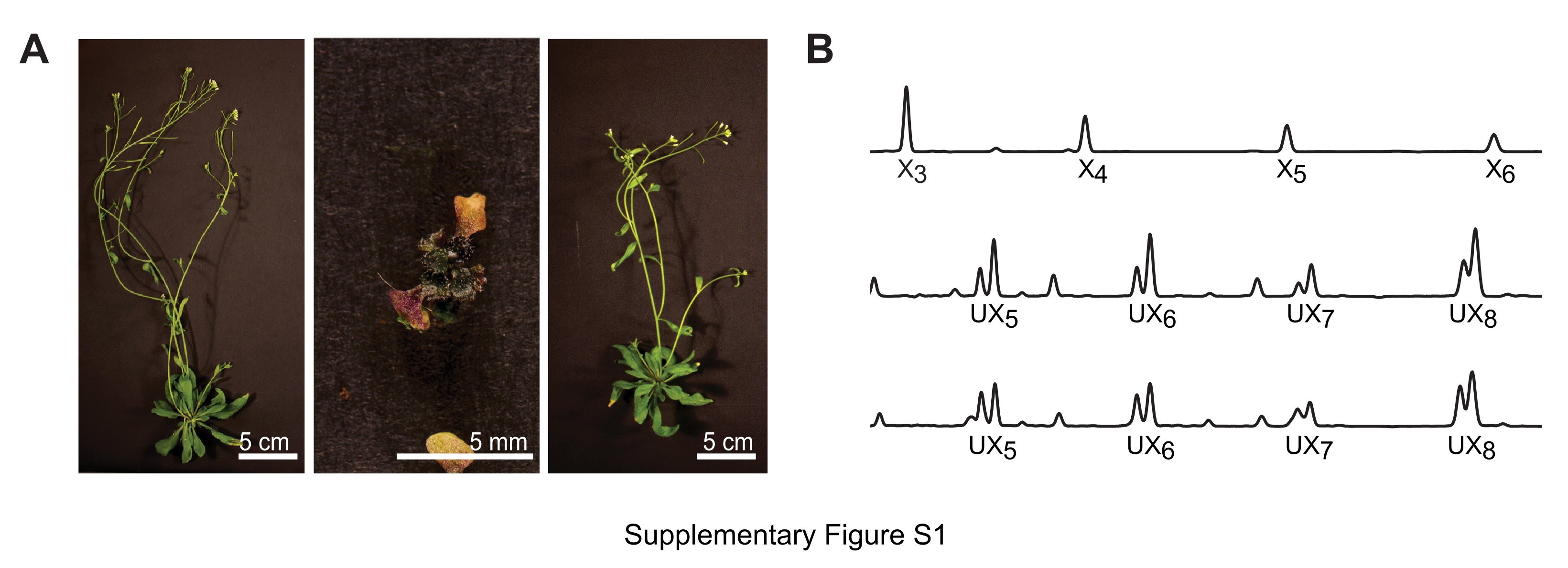

Supplement: Supplementary file 2 [file Image_1.jpg]

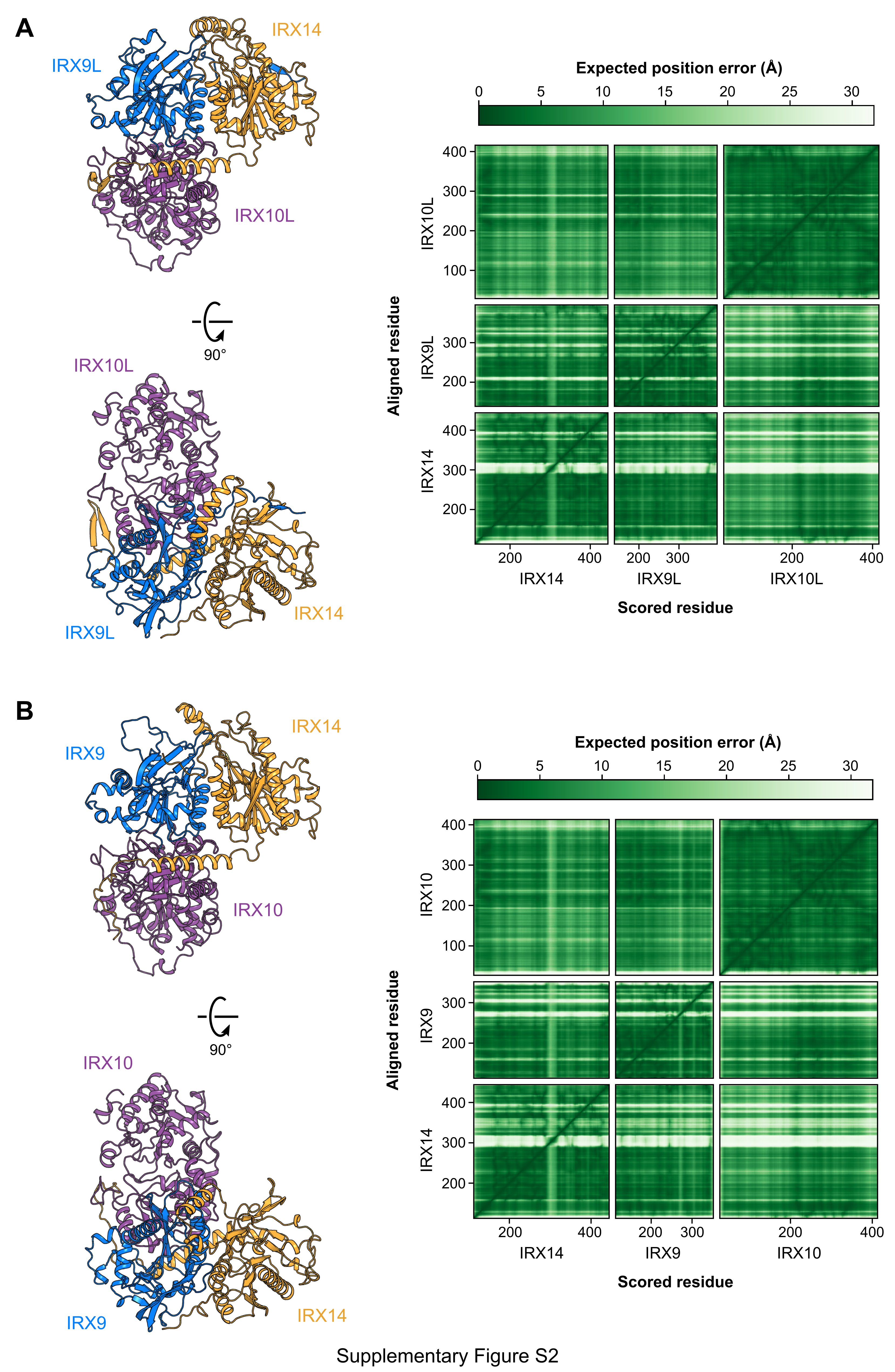

Supplement: Supplementary file 3 [file Image_2.jpg]

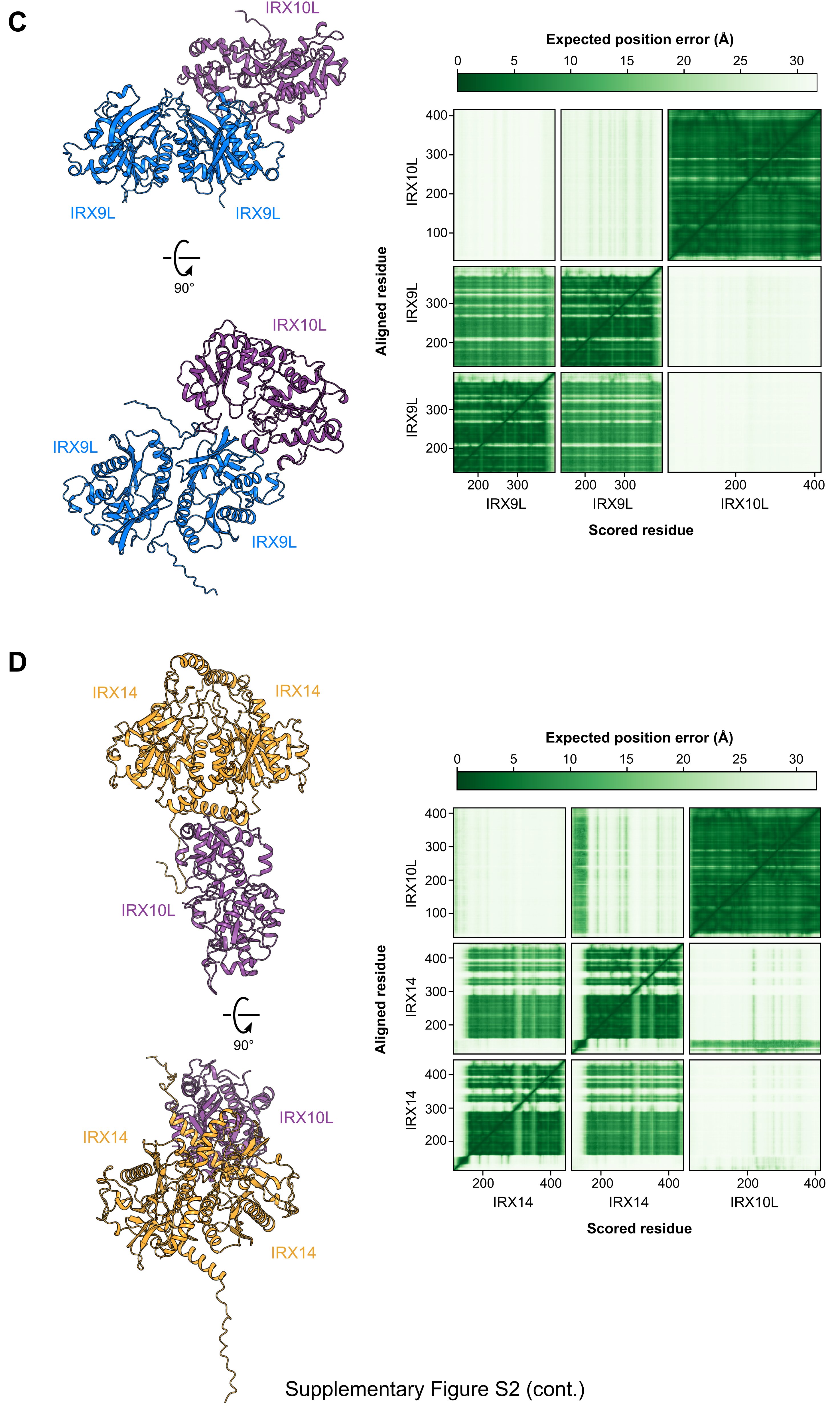

Supplement: Supplementary file 4 [file Image_3.jpg]

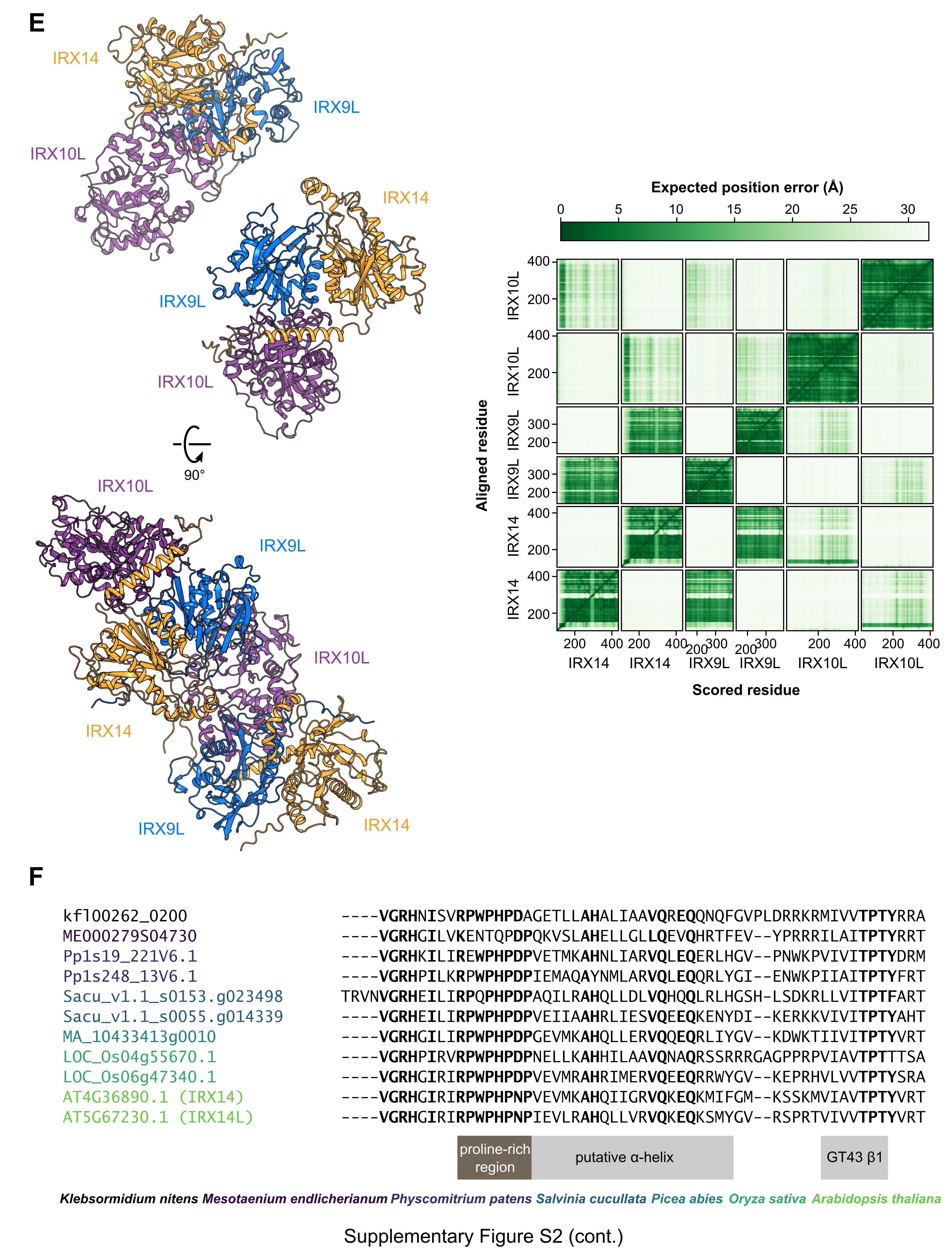

Supplement: Supplementary file 5 [file Image_4.jpg]

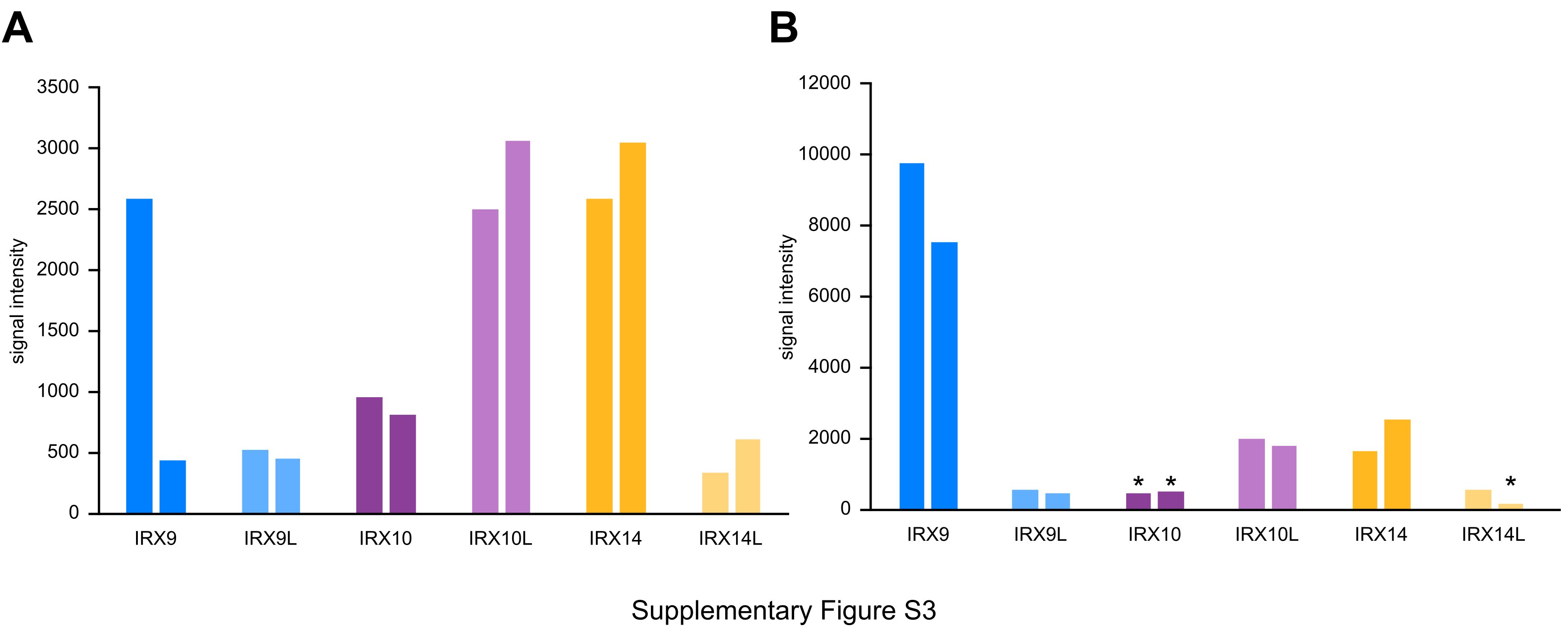

Supplement: Supplementary file 6 [file Image_5.jpg]
